# EZH2: novel therapeutic target for human cancer

**DOI:** 10.7603/s40681-014-0001-6

**Published:** 2014-02-12

**Authors:** Long-Yuan Li

**Affiliations:** 1 Graduate Institute of Cancer Biology, China Medical University, Taichung 404, Taichung, Taiwan; 2 Center for Molecular Medicine, China Medical University Hospital, Taichung 404, Taichung, Taiwan; 3 Department of Biotechnology, Asia University, Taichung 404, Taichung, Taiwan

**Keywords:** EZH2, Polycomb repressive, complex, Chromatin modification, Methylation

## Abstract

Enhancer of Zeste homlog 2 (EZH2) is a catalytic subunit of epigenetic regulator Polycomb repressive complex 2 (PRC2), which trimethylates Lys 27 of histone H3, leading to silencing of the target genes that are involved in a variety of biological processes including tumor progression and stem cell maintenance. However, in addition to its canonical PRC2-dependent transcriptional repression function, EZH2 also acts as a gene activator in a noncanonical PRC2-independent manner. Overexpression of EZH2 has been detected in diverse cancers, and is associated with tumor malignancy. Moreover, activating mutations and inactivating mutations of EZH2 are also associated with certain types of cancer. Given EZH2’s multi-faceted function and role in cancer, context-specific strategy for targeting EZH2/EZH2-mediated signaling could serve as future targeted therapy/personalized medicine for human cancer.

## Introduction

**Figure 1.**
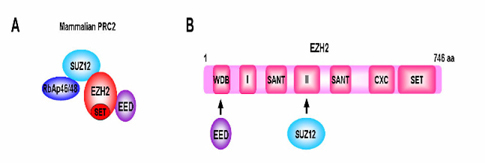
Core subunits of PRC2 and domain structure of EZH2. (A) Core subunits of mammalian PRC2 include suppressor of Zeste 12 (SUZ12), embryonic ectoderm development (EED), retinoblastoma suppressor-associated protein 46/48 (RbAp46/48), and histone methyltransferase (HMTase) EZH2, which catalyzes the trimethylation of histone H3 at Lys 27 (H3K27me3) to generate epigenetic silencing mark [10-14]. (B) Schematic diagram of EZH2 functional domains, WD-40 binding domain (WDB), domains I-II, two SWI3, ADA2, N-CoR and TFIIIB (SANT) domains, cysteine-rich CXC and evolutionarily conserved carboxy-terminal Su(var)3-9, enhancer of zeste, trithorax (SET) domain. EZH2’s SET catalytic domain is needed for HMTase activity [11, 14, 28, 29]. WDB is EED-interacting domain. Domain II is binding region for SUZ12, SANT domains for interaction with histone

Epigenetic regulation, including DNA methylation and demethylation [1]. histone modification, incorporation of histone variants [2, 3], and non-coding RNAs [4], plays a key role in modulating chromatin state and gene expression without altering DNA sequence. Aberrations of epigenetic regulators and chromatin modifications have proven links with human disease: e.g., cancer. Polycomb group proteins (PcGs) are crucial epigenetic regulators that form chromatin-modifying complexes, whose composition may be cell-context-dependent. In mammals, two major PcG complexes, Polycomb repressive complex 1 (PRC1) and 2 (PRC2), have been identified [5,6]. Core components of PRC1 complex contain ring finger protein RING1A/B, B lymphoma Mo-MLV insertion region 1 (BMI1), chromobox homolog (CBX), PHC, and SCML subunits [5-7]. The PRC1 establishes repressive chromatin structure via E3 ubiquitin ligase RING1A/B that monoubiquitylates Lys 119 of histone H2A (H2AK119ub1) [8, 9]. Core subunits of PRC2, conserved from Drosophila to mammals, include suppressor of Zeste 12 (SUZ12), embryonic ectoderm development (EED), retinoblastoma suppressor associated protein 46/48 (RbAp46/48), and histone methyltransferase (HMTase) EZH2, which catalyzes trimethylation of histone H3 at Lys 27 (H3K27me3) to generate another epigenetic silencing mark [10-14] (**Fig.** **1A**). However, study also reveals RING1B can maintain chromatin compaction and repress gene expression independent of its histone ubiquitination activity [15]. PcG proteins suppress transcription by forming chromatin loops with DNA methylation, which may impede DNA access to transcription factors [16]. Exact functions and molecular mechanisms underlying high-order chromatin configuration remain unexplored. Other than PcG proteins, Trithorax group (TrxG) proteins are known as critical to epigenetic regulation of senescence, cell cycle, DNA damage and stem cell biology. TrxG plays a role opposite to PcG: transcriptional activator of gene expression, modifying chromatin structure via trimethylation of histone H3 at Lys 4 (H3K4me3) [17]. It has been reported that many genes involved in development and differentiation concomitantly carry both repressive H3K27me3 and active H3K4me3 marks, known as bivalent chromatin domains. These bivalent loci are poised in a state ready for transcriptional activation or repression. Activated genes lose H3K27me3 and are enriched for H3K4me3; whereas repressed genes retain H3K27me3 or gain silencing marks like DNA methylation, but lose H3K4me3 [5].

**Figure 2.**
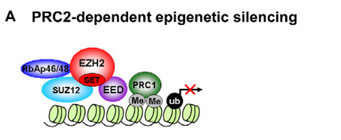

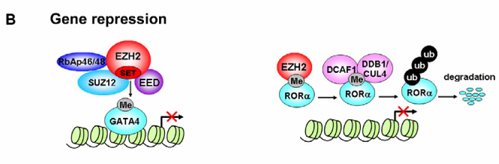

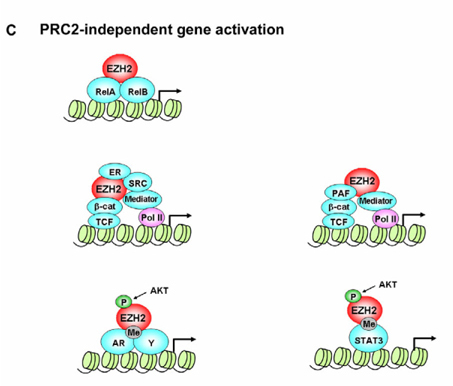
EZH2-mediated gene repression and activation mechanisms. (A) PRC2-dependent epigenetic silencing. With PRC2 (EZH2, EED, SUZ12, and RbAp46/48) recruited to target genes, EZH2 catalyzes trimethylation of histone H3 at Lys 27 (H3K27me3). PRC1 then recognizes and binds to H3K27me3 mark so as to monoubiquitylate Lys 119 of histone H2A (H2AK119ub1), resulting in epigenetic silencing. (B) EZH2-mediated gene repression via methylation of non-histone proteins. (Left) EZH2 interacts with and directly methylates cardiac transcription factor GATA4 at Lys 299. PRC2-mediated GATA4 methylation impairs interaction with and acetylation by p300, resulting in attenuated GATA4 transcriptional activity and gene repression [40]. (Right) EZH2 binds and methylates RORα at Lys 38 which is recognized and ubiquitinated by DCAF1/DDB1/CUL4 ligase complex, spawning RORα degradation and transcriptional repression of RORα target genes [41]. (C) PRC2-independent gene activation. (Top) EZH2 interacts with nuclear factor-kappaB (NF-κB) components RelA/RelB as ternary complex to activate gene expression [21]. (Middle left) EZH2 associates with ER and WNT signaling components TCF/β-catenin to activate ER target genes [20]. (Middle right) EZH2 forms complex with DNA repair protein PCNA-associated factor (PAF) and TCF/β-catenin to promote WNT target gene transactivation [18]. (Bottom left) AKT-mediated EZH2 phosphorylation at Ser21 promotes EZH2 binding with androgen receptor (AR) and methylating AR or AR-associated proteins, leading to transcriptional activation [22]. (Bottom right) EZH2 phosphorylation at Ser21 by AKT. Phosphorylated EZH2 interacts with and methylates STAT3 at Lys 180, augmenting STAT3 activity to yield transcriptional activation [19].

EZH2, catalytic subunit of PRC2, is predominantly considered to trimethylates Lys 27 of histone H3, leading to silencing of target genes involved in cell cycle regulation, senescence, cell fate determination, cell differentiation and cancer [6]. Yet besides its PRC2-dependent transcriptional repression function, recent evidence indicates that EZH2 also mediates gene activation through methylating nonhistone proteins or forming transcriptional complexes with other factors to activate downstream target genes in a PRC2-independent fashion [18-22] (**Fig.** **2**). Mounting evidence shows that overexpression/amplification or mutations of EZH2 have been detected in a variety of cancers, and are associated with tumor development and progression [23]. EZH2 also plays vital roles in stem cell maintenance and lineage differentiation [24-26]. Thus, EZH2/EZH2-mediated signaling deregulation contributes to numerous human pathologies, making this signaling an attractive therapeutic prospect and molecular marker to serve as targeted therapy/personalized treatment of human maladies, including cancers.

## Domain structure and function of EZH2

### Domain organization of EZH2

Human EZH2 was mapped to chromosome 7q35, which contains 20 exons and encodes 746 amino acids (**Fig.** **1B**) [27]. EZH2 harbors functional domains: WD-40 binding domain (WDB), domains I-II, two SWI3, ADA2, N-CoR and TFIIIB (SANT) domains, cysteine-rich CXC domain and evolutionarily conserved carboxy-terminal Su(var)3-9, enhancer of zeste, trithorax (SET) domain. The SET domain of EZH2 is the catalytic domain required for HMTase activity [11, 14, 28, 29]. Moreover, complex of EZH2 with other PRC2 components, noncatalytic subunits EED and SUZ12, is necessary to gain robust HMTase activity [28, 30-34]. WDB is EED-interacting domain. Domain II is binding region for SUZ12, and SANT domains are for interaction with histone.

### Polycomb-dependent transcriptional repression function of EZH2

As a catalytic subunit of PRC2, EZH2 is primarily considered as an epigenetic silencer for transcriptional repressing of gene expression, including a variety of tumor suppressor genes. With PRC2 recruited to target genes, EZH2 containing SET domain catalyzes trimethylation of histone H3 at Lys 27 (H3K27me3). Subsequently, the PRC1 subunit, CBX, recognizes and binds to H3K27me3 mark, then catalytic subunit of PRC1, RING1, monoubiquitylates Lys 119 of histone H2A (H2AK119ub1) to impede RNA polymerase II-dependent transcriptional elongation and repress gene transcription [5] (**Fig.** **2A**). Although coordinated recruitment of PRC1/2 is widely quoted in the literature, genome-wide mapping of histone modifications and localization of PRC1 and 2 subunits by chromatin immunoprecipitation-sequencing found that some genes that lack H2AK119ub1 are also targeted by PRC2 [35]. Besides, some reports indicate PRC1 recruitment and PRC1-mediated H2AK119ub1 occurring independently of PRC2/H3K27me3 [36-38]. Moreover, Tavares et al. recently unraveled that RYBP-PRC1 complex, consisting of RING1 YY1 binding protein (RYBP) and PRC1 catalytic subunits, mediates H2AK119ub1 at normal level both in PRC2-null mESCs and wild type mESCs, portending PRC2/H3K27me3 as not needed for recruitment of RYBP-PRC1 complex and RYBP-PRC1-mediated H2AK119ub1 on PcG target loci [39]. These studies revealed that PRC1 and PRC2 may also regulate gene expression independent of each other.

Aside from well-recognized epigenetic gene silencing function, recent study demonstrated EZH2 interacts with and directly methylates non-histone target, cardiac transcription factor GATA4 at Lys 299. PRC2-mediated GATA4 methylation impaired its interaction with and acetylation by p300 to attenuate GATA4 transcriptional activity and gene expression [40] (**Fig.** **2B**). Moreover, EZH2 binds and methylates RORα at Lys 38 which is recognized and ubiquitinated by DCAF1/DDB1/CUL4 ubiquitin ligase complex, giving rise to RORα degradation and transcriptional repression of RORα target genes [41] (**Fig.** **2B**). These studies explore novel mechanism of PRC2-mediated gene suppression: EZH2 directly methylating transcriptional factor and inhibiting transcriptional activity.

Diverse epigenetic modification regulators can cooperatively fine-tune gene expression. Indeed, EZH2 physically interacts with and recruits DNA methyltransferases DNMT1, DNMT3A and DNMT3B to methylate CpG and establish a more deeply repressive chromatin state [42]. Yet knocking down EZH2 increases transcription of genes with minimal DNA methylation but not genes with heavily DNA hypermethylation [43,44]. Several studies reveal EZH2 target genes pre-marked by PRC2-mediated H3K27me3 in normal cells as strongly correlated with genes becoming aberrantly hypermethylated in cancer cells, suggesting genes pre-marked H3K27me3 by PRC2 in normal development later become densely DNA-hypermethylated in the presence of oncogenic cues like abnormally elevated EZH2 expression [45-47]. Moreover, PRC2 interacts with histone deacetylase (HDAC), which may modify the histone mark by deacetylating H3K27 and relieving lysine side chain for methylation by PRC2, resulting in transcriptional silencing [48,49]. These three groups of epigenetic silencing regulators EZH2, DNMTs and HDACs may contribute to modulating aberrant gene expression in cancer cells, and their functional connections have been observed in cancers of colon, breast, lung, liver, ovarian and prostate, as well as acute promyelocytic leukemia [46,47,50].

### Polycomb-independent transcriptional activation function of EZH2

Apart form its transcriptional repression function, emerging studies uncover the noncanonical role of EZH2 showing that EZH2 also functions as an activator by methylating nonhistone proteins or forming transcriptional complexes with other factors to activate downstream target genes in a PRC2-independent manner [18-22] (**Fig.** **2C**). Lee et al. demonstrated that in estrogen receptor (ER)-negative basal-like breast cancer cells, EZH2 physically interacts with nuclear factor-kappaB (NF-κB) components RelA/RelB as a ternary complex to activate a subset of NF-κB target genes independently of its HMTase activity [21] (**Fig.** **2C**, top). In ER-positive luminal-like breast cancer cells, EZH2 also acts as an activator independently of its SET domain through association with ER and WNT signaling components TCF/β-catenin to activate ER target genes such as c-myc and cyclin D1 [20] (**Fig.** **2C**, middle left). Similarly, Jung et al. revealed that EZH2 forms complex with DNA repair protein PCNA-associated factor (PAF) and TCF/β-catenin to promote WNT target gene transactivation independently of EZH2’s HMTase activity, contributing to intestinal tumorigenesis [18] (**Fig.** **2C**, **middle right**).

In contrast to methyltransferase activity of EZH2 dispensable for EZH2-mediated gene activation mentioned above, Xu et al. demonstrated methyltransferase activity of EZH2 is required for both EZH2-mediated transcriptional activation and androgen-independent growth of castration-resistant prostate cancer cells [22]. AKT-mediated EZH2 phosphorylation at Ser21 promotes EZH2 binding with androgen receptor (AR) and methylating AR or AR-associated proteins, resulting in transcriptional activation of a subset of its target genes [22] (**Fig.** **2C**, **bottom left**). Recently, Kim et al. showed EZH2 phosphorylation at Ser21 by AKT is also required for EZH2 association with STAT3 and the enhanced STAT3 activity, that occur preferentially in glioma stem-like cells relative to non-stem tumor cells. Phosphorylated EZH2 interacts with and methylates STAT3 at Lys 180, which augments STAT3 activity by enhancing tyrosine phosphorylation of STAT3, resulting in transcriptional activation (**Fig.** **2C**, **bottom right**) [19]. Such results contrast with EZH2-mediated methylation of GATA4 or RORα, which decrease their transcriptional activity [40,41].

## Roles of EZH2 in cancer progression

Overexpression/amplification or mutation of EZH2 have been found in a wide range of cancer types, including breast, prostate, lung, liver, colon, ovarian, bladder, glioblastoma and lymphoma. Elevated expression of EZH2 correlates with tumor malignancy, poor prognosis and poor patient survival [6]. Overexpression and amplification of EZH2 is barely detected in early stage of prostate cancers, but is more general in late stages. Increased copies of EZH2, with corresponding enhanced protein expression, are found in more than 50% of the hormone-refractory prostate cancers [51]. Similarly, tissue microarray analysis of breast caner tissue samples showed EZH2 highly expressed in invasive breast cancer and metastatic breast cancer compared with normal or atypical hyperplasia, plus associated with breast cancer aggressiveness and poor clinical outcome [52]. Ecotopic expression of EZH2 in breast epithelial cells promotes oncogenic transformation by measuring anchorage-independent growth and cell invasion [52]. In EZH2 transgenic mouse model, ovexpression of EZH2 in mammary epithelial cells using the mouse mammary tumor virus long terminal repeat causes epithelial hyperplasia, highlighting the potential role of EZH2 in tumor progression [53].

Various heterozygous mutations of EZH2 at tyrosine 641 (Y641) in the SET domain have been found in 22% of germinal center B-cell and diffuse large B-cell lymphoma (DLBCLs) and 7% of follicular lymphoma [54]. This Y641 somatic mutation causes a gain-of-function mutation, but Y641 mutant EZH2 exhibits catalytic activity toward substrates differently from wild-type (WT) EZH2. Y641 mutant EZH2 preferentially catalyzes tri-methylation of H3K27, but exhibits limited ability to catalyze the first mono- and di-methylation of H3K27. By contrast, WT EZH2 exerts highest catalytic activity for the first monoand di-methylation of H3K27, but relatively weak catalytic activity for the tri-methylation of H3K27 [55]. Intriguingly, Y641 mutant EZH2 detected in B-cell lymphoma is always heterozygous. Thus, heterozygous Y641 mutant EZH2 can work along with WT EZH2 to raise H3K27me3 levels, which may be functionally like EZH2 overexpression [56]. Another heterozygous mutation of EZH2 at alanine 677 to glycine (A677G) is also identified in lymphoma cell lines and primary lymphoma samples with frequency less than 2-3% [55]. Similar to activating mutation of Y641 mutant EZH2, expression of A677G mutant EZH2 induces a global hypertrimethylation of H3K27. However, different from WT EZH2 and Y641 mutant, A677G mutant EZH2 efficiently methylates all three substrates (unmodified, mono- and dimethylated H3K27), indicating A677G mutant EZH2 deregulates H3K27 methylation without needing working with WT EZH2 as is the case for Y641 mutant EZH2 [55].

Mutations of EZH2 are detected in 10-13% of myelodyplasia-myeloproliferative neoplasm, 13% of myelofibrosis and 6% of various myelodysplastic syndrome subtypes, not occurring at single residue but throughout the gene. Most such mutations are nonsense and stop codon mutation, resulting in loss of HMTase activity, apparently unlike Y641 and A677 mutants [57], raising the possibility for EZH2 acting as a tumor suppressor. These studies indicate both activating and inactivating mutations of EZH2 can be associated with certain malignancy, their differential roles in regulating specific cohort of target genes that contribute to tumorigenesis may be context dependent and need to be explored further.

## Targeting EZH2 or EZH2-mediated signaling for potential cancer therapy

Given its role in tumor progression and stem cell maintenance, EZH2 or EZH2-mediated signaling may be attractive targets for potential cancer therapeutics. Several studies show the small molecule 3-deazaneplanocin A (DZNep), a S-adenosylhomocysteine hydrolase inhibitor, which inhibits methylation reaction and induces EZH2 degradation, suppresses various types of cancer growth and reduce tumor formation: e.g., glioblastoma cancer stem cells, ovarian cancer stem cell-like populations, prostate cancer/cancer stem cells [23]. Still, DZNep is not a specific EZH2 inhibitor; it also influences other processes that involve methylation reaction. Recently, several highly potent and selective inhibitors of EZH2, such as GSK126, GSK343, EPZ005687, EPZ-6438 and El1, have been discovered [58]. Among them, GSK126 and EPZ-6438 are the most potent and selective S-adenosyl-methionine (SAM)-competitive, small-molecule inhibitors of EZH2 methyltransferase activity. GSK126 could effectively suppress proliferation of EZH2 mutant DLBCL cell lines and inhibit tumor growth in xenograft mouse model of EZH2 mutant DLBCL in vivo, indicating pharmacological inhibition of EZH2 activity may show promise in treating DLBCL harboring activating mutations of EZH2 [59]. EPZ-6438 causes apoptosis and differentiation in SMARCB1 mutant malignant rhabdoid tumor (MRT) Cells, and completely inhibits growth of MRT xenografts in mice without tumor regrowth after dosing cessation [60]. This study reveals that inhibition of EZH2 activity may be a compelling therapeutics for a spectrum of cancers with genetic alterations conferring a proliferative dependency on EZH2 enzymatic activity despite EZH2 itself is not genetically changed in the cancers. EPZ-6438 also eliminates the growth of several EZH2 mutant xenografts including WSU-DLCL2 (Y641F), Pfeiffer (A677G), KARPAS-422 (Y641N) etc., and has been approved for human clinical trials in patients with advanced solid tumors or with B-cell lymphoma [58]. Moreover, down regulation of EZH2 expression by siRNAs or shRNAs has also been shown to inhibit cancer cell and tumor growth [23]. Besides direct blocking EZH2 activity/expression, EZH2-mediated tumorigenic signaling is another attractive therapeutic target. For example, study shows EZH2 up-regulating RAF1-ERK-β-catenin pathway, leading to promoting survival and proliferation of breast tumor initiating cells [23]. Inhibitors of RAF1-ERK signaling, such as sorafenib and AZD6244, are plausible therapeutic agents to eradicate breast tumor initiating cells.

## Perspectives

EZH2, deregulated in a wide range of cancers, exerts its functions in distinct action modes (**Fig.** **2**). Functioning in both PRC2-dependent (canonical) and -independent (noncanonical) manners to repress or activate target gene expression, it thus may contribute to tumorigenesis via both positive and negative regulation of gene activity in cell-context dependent manner. Currently, no EZH2 inhibitors are approved for treatment of human cancers; much effort has been made to develop EZH2 HMTase inhibitors. Since methyltransferase activity of EZH2 is not required for certain EZH2-mediated gene activation (**Fig.** **2C**), alternative strategy aside from inhibiting EZH2 enzymatic activity warrants attention. In this regard, approaches based on disrupting interaction between EZH2 and other factors like ER/TCF/β-catenin and RelA/RelB might be potential therapeutic targets.

In addition to overexpression of EZH2 in cancers, activating mutations and inactivating mutations of EZH2 correlate with certain types of cancer, pointing to the complicated role of EZH2 mutants in cancer meriting further exploration: e.g., whether gain of EZH2 function mutant modulates a set of genes similar to or different from those regulated by inactivating mutation of EZH2. Given EZH2’s multi-faceted role in cancer, insight into sophisticated regulatory mechanisms of EZH2/EZH2-mediated signaling will pave the way for developing context- or allele-specific (mutant EZH2-specific) strategy for targeting EZH2/EZH2-mediated signaling that could serve as future targeted therapy/personalized medicine for human cancer.

## Acknowledgements

This work was supported by grants from National Science Council (NSC102-2321-B-039-002, NSC102-2325-B-039-002, NSC99-2632-B- 039-001-MY3 to L.-Y.L.) and the Ministry of Health & Welfare (MOHW102-TD-PB-111-NSC105 to L.-Y.L.).
